# Pulmonary embolism in COVID-19, risk factors and association with inflammatory biomarkers

**DOI:** 10.1097/MD.0000000000032887

**Published:** 2023-02-17

**Authors:** Muhammad Yousaf, Merlin Marry Thomas, Salah Almughalles, Mansoor Ali Hameed, Ahmad Alharafsheh, Irfan Varikkodan, Ali Waseem, Mona Babikir, Dinesh Chengamaraju, Mohamad Yahya Khatib

**Affiliations:** a Hazm Mebaireek General Hospital, Hamad Medical Corporation, Doha, Qatar; b Weill Cornell Medicine Qatar, Cornell University, Doha, Qatar; c Hamad General Hospital, Hamad Medical Corporation, Doha, Qatar.

**Keywords:** COVID-19, critical illness, CTPA, D-Dimer, pulmonary embolism, venous thromboembolism

## Abstract

The coronavirus disease 2019 (COVID-19) pandemic affected millions of people worldwide resulting in a substantial number of hospitalizations. Venous thromboembolism including pulmonary embolism is a known complication of COVID-19 pneumonia although its incidence in such patients is unclear. In this multicenter retrospective cohort study, we looked at the incidence of pulmonary embolism in COVID-19 patients and its associations with various risk factors including demographics, comorbidities, inflammatory markers and coagulation profiles. We analyzed data from 193 patients of mixed ethnicity with a mean age of 51, mostly South Asians (62%) and Arabs (29%). Diabetes and hypertension were the most prevalent comorbidities accounting for 46% (N = 88) and 36% (N = 71) respectively. Critical COVID-19 illness was diagnosed in 67% of patients. The frequency of COVID-19 related pulmonary embolism was 21.8% (N = 42). We found no association of pulmonary embolism with demographic, comorbid or inflammatory variables. Only a raised D-Dimer was found to be associated with pulmonary embolism. Having a pulmonary embolism had no impact on the length of stay, critical illness, or mortality. Receiving steroids or being on standard thromboprophylaxis or weight/D-Dimer adjusted thromboprophylaxis also had no impact on the frequency of pulmonary embolism. Nine incidents of major bleeding were recorded independent of therapeutic anticoagulation. Patients admitted to the hospital for COVID-19 pneumonia had a relatively high incidence of pulmonary embolism. D-dimer was the only associated laboratory parameter associated with pulmonary embolism. However, further research is needed to evaluate its predictive and prognostic utility, particularly in an older population.

## 1. Introduction

The coronavirus disease 2019 (COVID-19) pandemic has afflicted millions of people worldwide and poses an unprecedented challenge to public health, costing human life and the global economy.^[1]^ Venous thromboembolism is a recognized complication of COVID-19, in fact, many studies have reported a higher incidence of deep vein thrombosis and pulmonary embolism (PE) in COVID-19.^[2,3]^ Importantly, PE in COVID-19 has been found to be different from traditional PE in non-COVID medical patients in terms of demographics, clinical, and laboratory characteristics.^[4]^ Emerging research on the pathophysiology of PE in COVID-19 has suggested the concept of pulmonary microvascular thrombosis rather than the classic venous thrombus dislodging and traveling as an embolus to pulmonary vasculature,^[5,6]^ suggesting that the risk factors for PE in COVID-19 patients may differ from those in non-COVID patients. Thromboembolic complications of COVID-19 remain a topic for hot debate and the primary aim of our study was to determine the incidence and risk factors of pulmonary embolism in COVID-19 patients admitted to covid hospitals in the state of Qatar.

## 2. Materials and methods

### 2.1. Study design, setting and population

We conducted a retrospective observational study from March 1, 2020 to July 20, 2020 among COVID-19 patients with suspected pulmonary embolism (PE) who were admitted to the 3 Covid-19 hospitals of Hamad medical corporation. The 3 hospitals included Hazm Mebaireek general hospital, communicable disease center and Cuban hospital.

### 2.2. Data collection

The study included all the patients hospitalized to Hamad medical corporation 3 Covid-19 facilities who underwent a Computed tomography pulmonary angiography scan (CTPA) for suspected pulmonary embolism (PE). This data was collected using digital searches on Synapse Clinical Imaging System (SYNAPSE PACS) for all CTPA done during the study period. Based on the presence or absence of pulmonary embolism on the CTPA, the patients were categorized as cases or controls. Any patient over the age of 18 with a severe acute respiratory syndrome coronavirus 2 positive test by reverse transcriptase polymerase chain reaction in a nasopharyngeal or oropharyngeal swab was included. The exclusion criteria included previous venous thromboembolism, underlying malignancy, pregnancy and being on therapeutic anticoagulation prior to admission. Data collected to address the study objectives included demographics, comorbidities, length of stay, and mortality and use of anticoagulants and steroids. In addition, data was collected on inflammatory, biochemical and coagulation parameters, thrombotic events, bleeding complications and acute kidney Injury. Furthermore, we reviewed the data on critical care admissions, and ventilatory support and incidence of acute respiratory distress syndrome.

### 2.3. Outcomes and definitions

The primary aim of our study was to identify an association of PE with inflammatory biomarkers (interleukin-6 [IL-6], C-reactive protein [CRP], Ferritin, Troponin, lactate dehydrogenase [LDH]), and lymphopenia. The secondary objectives were to explore an association of PE with diabetes, body mass index (BMI) and coagulation parameters (D-Dimer, Platelets, international normalized ratio) and to determine its incidence and effect on mortality.

Pulmonary embolism (PE) was defined as radiologically confirmed pulmonary embolism on CTPA. The major and minor bleeding complications were defined as per the International Society on Thrombosis and Haemostasis (ISTH).^[7]^ Critical illness was defined as that required either inotropic, organ (e.g., Dialysis) or ventilatory support including noninvasive ventilation. The Acute respiratory distress syndrome was defined by Berlin 2012 Criteria.^[8]^ Acute kidney injury was defined as creatinine rise > 1.5 times or > 26.5 umol/L from the baseline.^[9]^ Severe COVID pneumonia was defined as pneumonia that was associated with hypoxemia on room air^[10]^

The South Asians in our study refer to people from India, Bangladesh, Nepal, Pakistan, and Sri Lanka. Southeast Asians refer to people from countries such as the Philippines, Brunei, Cambodia, Indonesia, Laos, Malaysia, Myanmar, and Singapore. The Philippines accounted for the majority of Southeast Asians in our study. In addition to the local Arab population, the Arabs included people from other Arabic-speaking countries, including Egypt, Sudan, Libya, Lebanon, Syria, and Iraq.

### 2.4. Inflammatory and coagulation parameters

We identified initial (or baseline) lab parameters as those done within 48 hours of admission and peak parameters as the highest value recorded during the hospital course. Coagulation and hematology profile included international normalized ratio international normalized ratio, activated partial thromboplastin time, platelets, D-Dimer, and lymphocyte count. The biochemical markers included calcium, HbA1c, and high-sensitivity cardiac troponin. The inflammatory parameters included CRP, Ferritin, LDH, and IL-6.

### 2.5. Statistical analysis

Descriptive statistics were used to summarize the sample characteristics and distribution of various considered variables related to demographics, clinical profiles and laboratory parameters. The normally distributed data and results were reported with mean and standard deviation (SD) with corresponding 95% confidence interval. Categorical data were summarized using frequencies and percentages. The correlation between the quantitative outcome measures (derived using different surveys) were computed using Pearson or Spearman rank correlation coefficient (r) wherever applicable. Association between various categorical variables of clinical data and outcome measures were assessed using Pearson Chi-square or Fisher Exact tests as appropriate and *t* test or analysis of variance for quantitative outcome measures. Univariate and multivariate logistic regression methods were used to assess the association of risk factors to the incidence of PE in COVID-19. For multivariate regression models, variables were considered if statistical *P* < .05 level at univariate analysis or if determined to be clinically important. *P* < =.05 (2 tailed) was considered statistically significant. Statistical package SPSS 28.0 (SPSS Inc. Chicago, IL) and Epi Info 2000 (Centres for Disease Control and Prevention, Atlanta, GA) was used for the analysis.

## 3. Outcome and results

Off the 230 initially screened patients, 37 were excluded primarily due to incomplete or missing essential data. Hence, our study included a total of 193 patients with a mean age of 51, a relatively younger population with 78% of patients < 60 years of age. Our study population had mixed ethnicity and predominantly comprised of South Asians (62%) and Arabs (29%). Diabetes was shown to be the most prevalent comorbidity (45% (n = 88), followed by hypertension (36% (n = 71). At admission, the majority (55%) of the patients had severe COVID-19 Pneumonia (hypoxemic). Table 1 lists the baseline characteristics of the patients grouped into 2 cohorts based on the presence or absence of a primary outcome event that is, PE.

**Table 1:** Baseline characteristics of COVID-19 hospitalized patients.

Baseline characteristics	Total patients n = 193	PE n = 42 (21.8%)	No PE n = 151 (78.2%)	*P* value
**Age, mean ± SD, yr**	51.48 ± 2.8	50.5 ± 12.4	51.8 ± 12.9	
≤ 60 yr	151 (78.2)	33(78.6)	118(78.1)	.95
>60 yr	42 (21.8)	9 (21.4)	33 (21.9)	
**Gender**				.38
Male	184 (95.3)	39 (92.9)	145 (96)	
Female	9 (4.7)	3(7.1)	6 (4)	
**Ethnicity**				.012
South Asian	121 (62.7)	27 (64.3)	94 (62.3)	
Arab	29 (29)	4 (9.5)	25 (16.6)	
Southeast Asian	29 (15.0)	4 (9.5)	25 (16.6)	
African	9 (4.7)	6 (14.3)	3 (2.0)	
Others	5 (2.6)	1 (2.4)	4 (2.6)	
**Smoking**				.85
Never smoker	90 (46.6)	20 (47.6)	70 (46.4)	
Smoker	28 (14.5)	7 (16.7)	21 (13.9)	
Unknown	75 (38.9)	15 (35.7)	60 (39.7)	
**BMI**				.14
Normal (18.5-24.9)	49 (26.9)	16 (39)	33 (23.4)	
Overweight (25-29.9)	80 (44)	15 (36.6)	65 (46.1)	
Obese (≥ 30)	53 (29.1)	10 (24.4)	43 (30.5)	
**Comorbidities**				
Diabetes	88 (45.6)	18 (42.9)	70 (46.4)	.68
Hypertension	71 (36.8)	12 (28.6)	59 (39.1)	.21
Ischemic heart disease	13 (6.7)	3 (7.1)	10 (6.6)	.91
Chronic kidney disease	2 (1.0)	0	2 (1.3)	.45
Asthma	10 (5.2)	1 (2.4)	9 (6)	.36
Tuberculosis	3 (1.6)	0	3 (2)	.36
Asthma	10 (5.2)	1 (2.4)	9 (6)	.36
Cerebrovascular disease	5 (2.6)	1 (2.4)	4 (2.6)	.92
Malignancy	3 (1.6)	0	3 (2)	.36

BMI = body mass index, PE = pulmonary embolism.

The incidence of confirmed PE was 21.8% (n = 42) in all patients screened with CTPA during the study period. Average length of stay (LOS) of our cohort was 38.64 ± 40.6 days. A relatively longer LOS observed, in our study, likely reflects a higher proportion of critically unwell patients 67% (n = 130). However, having a PE did not increase the chance of critical illness. Table 2 presents the clinical characteristics of COVID-19 Hospitalized patients based on the presence or absence of PE.

**Table 2:** Clinical characteristics of COVID-19 hospitalized patients.

Clinical characteristics	Total patients n = 193	PE n = 42 (21.8%)	No PE n = 151 (78.2%)	*P* value
**Hospitalization status**				
Non-hypoxic on admission	87 (45.1)	16 (41)	71 (49.7)	.34
Non-critical illness	63 (32.6)	11 (26.2)	52 (34.4)	
Critical illness	130 (67.4)	31 (73.8)	99 (65.6)	.31
noninvasive ventilation	73(37.8)	22 (52.4)	51 (33.8)	.028
Mechanical ventilation	98 (50.8)	21 (50)	77 (51.0)	.91
ARDS	106 (54.9)	24 (57.1)	82 (54.3)	.74
Length of stay	38.64 ± 40.6			
Deceased	38 (19.7)	7 (16.7)	31(20.5)	.57
**Other clinical parameters**				
Steroid course	106 (54.9)	23(54.8)	83(55)	.98
Acute kidney injury	72 (37.3)	13 (31)	59 (39.1)	.34
Co-infection	88 (45.6)	20 (47.6)	68 (45)	.86
**Bleeding and coagulation**				
Bleeding complications	20 (10.4)	3 (7.1)	17 (11.3)	.43
Major bleeding evets	9	1	8	.42
Antiplatelets	31 (16.1)	7 (16.7)	24(15.9)	.9
Anticoagulation prophylaxis	187 (96.9)	40 (95.2)	147 (97.4)	.48

PE = pulmonary embolism.

In our analysis we found no association between PE and inflammatory biomarkers (IL-6, CRP, Troponin, LDH, Ferritin) or lymphopenia in COVID-19 patients (Table 3). The most prevalent comorbidity observed was diabetes 45% (n = 88) and it showed no correlation to PE. Most patients had a higher BMI (73%) either being overweight (44%) or obese (29%), however, a higher BMI did not increase the risk of getting a PE.

**Table 3:** Laboratory parameters of COVID-19 hospitalized patients.

Laboratory parameters	Mean ± SD	PE n = 42 (21.8%)	No PE n = 151 (78.2%)	*P* value
**Lymphocyte**, x10^3/uL				
Initial (Baseline)	2.3 ± 3.1	2.3 ± 3.3	2.3 ± 3.1	.83
Min	5.5 ± 8.5	1.3 ± 1.9	1.3 ± 1.9	.87
**Platelet** x10^3/uL				
Initial (Baseline)	225.4 ± 90.8	235.8 ± 92.6	222.5 ± 90.3	.41
Peak	482 ± 161.8	468.7 ± 149	486.2 ± 165.6	.53
**D-Dimer** mg/L				
Initial (Baseline)	3.6 ± 11.8	7.4 ± 22.3	2.7 ± 7.0	.03
Peak	22.2 ± 27.8	39.1 ± 36.5	17.2 ± 22.7	.00
**IL-6**				
Initial (Baseline)	212 ± 701	48.8 ± 37	260 ± 792	.22
Peak	1638 ± 5150	895.2 ± 1184.6	1813 ± 5689	.42
**CRP** mg/L				
Initial (Baseline)	129.7 ± 93	123.5 ± 71.4	131 ± 98.2	.64
Peak	234 ± 122.5	231.1 ± 124.6	234.9 ± 122.3	.86
**Ferritin** ug/L				
Initial (Baseline)	1750 ± 4080	1427 ± 1719	1842 ± 4530	.59
Peak	6156 ± 13940	5952 ± 15237	6215.2 ± 13602	.91
**LDH** U/L				
Initial (Baseline)	522.5 ± 207	565.8 ± 192.1	511.7 ± 211	.28
Peak	784 ± 383	868.6 ± 448.9	759.2 ± 360	.13
**Troponin** ng/L				
Initial (Baseline)	26.2 ± 75	18.8 ± 33.7	27.8 ± 81.3	.63
Peak	157.8 ± 478.9	164.3 ± 393.7	156.2 ± 498	.93
**Calcium** Peak mmol/L	2.5 ± 0.24	2.6 ± 0.2	2.6 ± 0.3	.84
**HbA1c** %	7.6 ± 3	7.7 ± 2.6	7.6 ± 3.1	.83
**INR**	1.16 ± 0.4	1.1 ± 0.1	1.2 ± 0.4	.28
**APTT** seconds	31.3 ± 4.8	29.9 ± 4.2	31.6 ± 4.9	.04

APTT = activated partial thromboplastin time, CRP = C-reactive protein, IL-6 = interleukin-6, INR = international normalized ratio, LDH = lactate dehydrogenase, PE = pulmonary embolism.

Among coagulation parameters, only D-Dimer was clearly associated with PE as would be expected. Raised D-Dimer, whether at admission (baseline) or during the course of illness (peak value) showed a statistically significant association with PE.

All the patients who had PE (n = 42) were on standard dose prophylactic anticoagulation except 2. Among the patients who had PE, 7 were on antiplatelets prior to the hospital admission. Being on antiplatelet did not confer protection against PE, however, the numbers were too small to draw any meaningful conclusions.

Having pulmonary embolism had no impact on the LOS, critical illness or mortality. Overall, 38 patients (19.7%) died with 31 deaths in non-PE group as opposed to 7 deaths in PE group (*P* = .57). As expected, critical illness was associated with statistically significant higher mortality (*P* < .001), all deaths occurred in critically unwell except 1 (Table 4). Hypertension (*P* = .003) and AKI (P0.001) were the only 2 comorbid conditions that were associated with falling critically ill. Although PE did not show a correlation with critical illness, however, patients with PE were more likely to require noninvasive ventilation support 52% (n = 22) as opposed to 33% (n = 51) in non-PE group (P < .028). The old age (>60 years) did not increase the chance of having critical illness or increased mortality.

**Table 4:** Critical illness in COVID-19 hospitalized patients.

Variables	Total patients n = 193	Critical illness (n = 130)	No critical illness (n = 63)	*P* Value
**Age, mean ± SD, yr**				.08
≤ 60 yr	151 (78.2)	97 (74.6)	54 (85.7)	
>60 yr	42 (21.8)	33 (25.4)	9 (14.3)	
**Comorbidities**				
Diabetes	88 (45.6)	57 (43.8)	31 (49.2)	.48
Hypertension	71 (36.8)	57 (43.8)	14 (22.2)	.003
Ischemic heart disease	13 (6.7)	11 (8.5)	2 (3.2)	.16
AKI	72 (37.3)	64 (49.2)	8 (12.7)	<.001
Asthma	10 (5.2)	8 (6.2)	2 (3.2)	.38
**Complications**				
Bleeding complications	20 (10.4)	18 (13.8)	2 (3.2)	.02
Death	38 (19.7)	37 (28.5)	1 (1.6)	<.001

COVID-19 = coronavirus disease 2019.

Majority of critically unwell patients 54.9% (n = 106) received a minimum of 5 days course of steroids prior to a diagnosis of PE. For critically ill covid patients our intensive care protocol used methylprednisolone 40mg TDS for 10 days. However, our results show receiving a course of steroids did not influence the occurrence of PE (*P* = .98).

A total of 20 patients (10.4%) experienced a bleeding event, with 9 experiencing a major bleeding event. Being on therapeutic anticoagulation or having a PE, however, had no effect on the incidence of bleeding complications. On review of case notes for the minor bleeding events, although a drop in hemoglobin was documented, but a distinct bleeding episode could not be identified. This discovery casts doubt on the veracity of the reported minor bleeding occurrences.

## 4. Discussion

Our observational study describes the baseline characteristics of COVID-19 patients with pulmonary embolism and evolution of inflammatory and biochemical parameters during the course of hospitalization. We found that old age, obesity, diabetes, hypertension, prior VTE, and smoking were not associated with a higher incidence of PE, thus supporting the findings of previous studies that the traditional risk factors for VTE were not associated with the occurrence of PE in COVID-19,^[11]^ though the results of those studies are conflicting.

**Figure 1.**
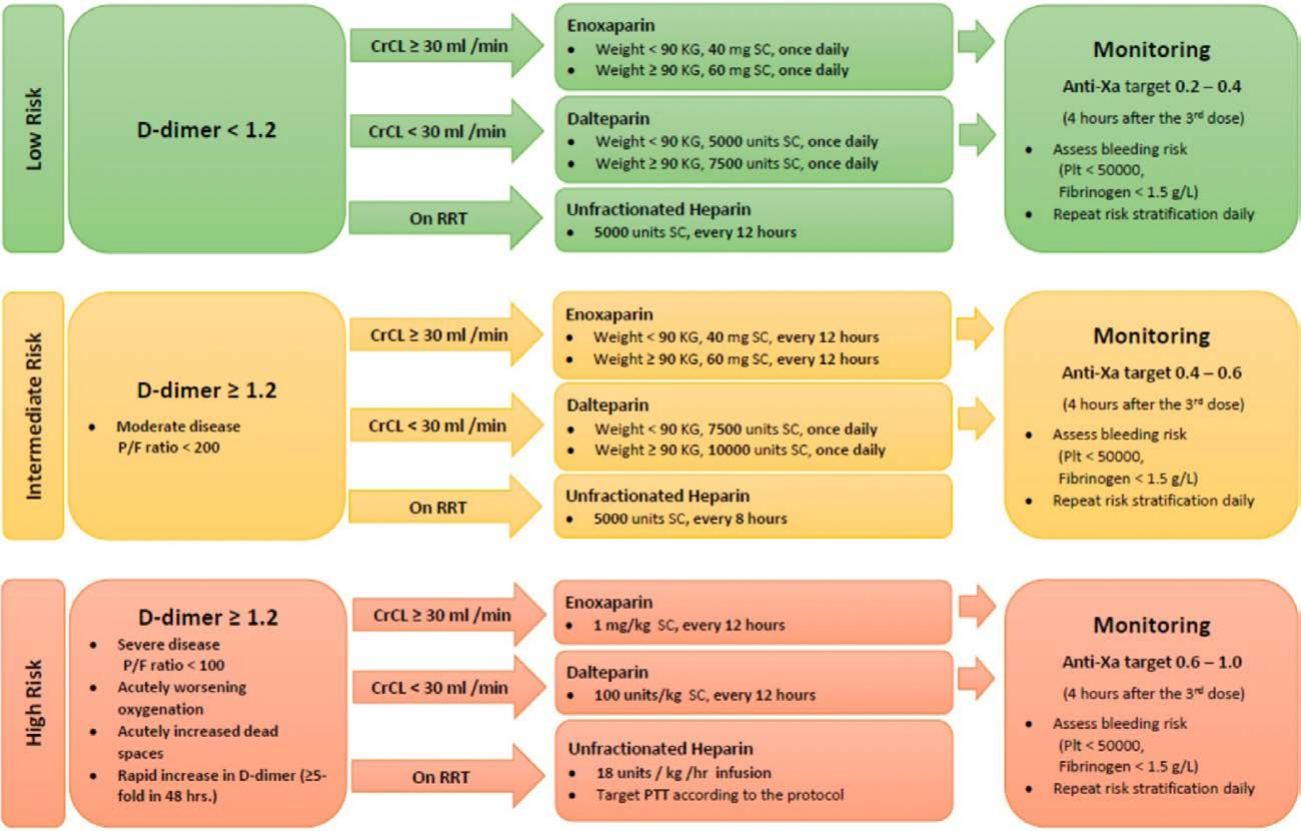
Weight adjusted, and D-Dimer based prophylactic anticoagulation protocol.

We reported an incidence of 21.8% for radiologically confirmed PE in COVID-19 hospitalized patients, of which 67% patients were critically unwell. Our study indicates a higher occurrence of PE in COVID-19, a finding in line with the results of previous studies with a range of 5% to 31% incidence reported.^[12,13]^ In our COVID-19 facilities we used thromboprophylaxis for all COVID-19 patients admitted to hospital. Our local thromboprophylaxis protocol included standard dose prophylaxis and higher dose regimen adjusted for weight and D-dimer (Fig. 1). However, despite adequate thromboprophylaxis the incidence of PE remained significantly higher compared to what would be expected in non-COVID medical patients.^[14,15]^ It should be noted, though, that the majority of our study population was critically unwell and critical illness is a recognized risk factor for VTE.^[16]^ In addition, we reported the incidence of PE among patients with suspected PE that is a select high risk group. On the other hand, a lack of uniform protocol for investigating suspected PE may have led to underestimation of the occurrence of PE in our study. A higher prevalence of PE in COVID-19, despite adequate thromboprophylaxis, is not unusual and has been reported by other studies^[17]^

No significant difference was observed in demographics, comorbidities, inflammatory markers, and coagulation parameters in COVID-19 patients with regards to occurrence of PE, an observation similar to other studies^[18,19]^

We looked at a wide range of inflammatory biomarkers and laboratory parameters to ascertain an association with PE in COVID-19. Our study found no correlation of PE to various laboratory parameters including CRP, IL-6, Troponin, LDH, ferritin, and lymphopenia. Although, Hanny Al-Samkari, and Colleagues found an association of VTE with inflammatory biomarkers,^[20]^ however, their study had far fewer critically unwell patients (36%) than our study (67%). Many other studies had looked at various risk factors including demographics, comorbidities and lab parameters for VTE in COVID-19 and have produced inconsistent and conflicting results.^[11,21,22]^

Various other studies have reported a strong correlation of raised D-dimer with the incidence of VTE in COVID-19, as demonstrated by Tan and colleagues in a meta-analysis^[23]^ and our findings are consistent with those studies.

The majority of our critical unwell COVID-19 patients received high-dose steroids. Later, RECOVERY trial demonstrated a mortality benefit with the use of steroids in severe COVID-19 pneumonia.^[24]^ We assumed that the use of an anti-inflammatory agents like steroids may dampen the inflammation induced endotheliopathy and thus thrombotic complications of COVID-19. In our study, however, having a course of steroids had no effect on the incidence of PE. In contrast, Ronaldo and colleagues found an increase in VTE in individuals on steroids in their study.^[25]^

The limitations of our study as a retrospective observational design are evident. We only looked at VTE and not COVID-19 related arterial thrombotic events such as myocardial infarctions or strokes. Our results must be interpreted with caution, as they could be biased by the particular circumstances of the first pandemic wave, including the high number of CTPA requested in COVID-19 pneumonia, making the diagnosis of PE more frequent and easier. Our study population comprised of 95% male and that reflects dynamics of the first pandemic wave in Qatar and demographics of the local population in general. Our study as opposed to western studies had relatively younger population and a unique mix of ethnicities including predominantly South Asians and Arab population. Nonetheless, our results indicate a higher prevalence of pulmonary embolism in hospitalized COVID-19 patients despite adequate thromboprophylaxis and an association with raised D-Dimer.

## 5. Conclusion

Despite its limitation, our study proves a higher incidence of PE in COVID-19 that does not appear to be associated with the traditional risk factors for PE. It seems that the jury is still out when it comes to defining the risk factors for PE in COVID-19 as various studies have reported conflicting results. D-dimer, on the other hand, has demonstrated an association with PE in COVID-19, however, its predictive role and prognostic value in PE, critical illness and death in COVID-19 remain to be ascertained.

## Author contributions

**Conceptualization:** Muhammad Yousaf, Merlin Marry Thomas, Mansoor Ali Hameed.

**Data curation:** Salah Almughalles, Ahmad Alharafsheh, Irfan Varikkodan, Ali Waseem, Mona Babikir, Dinesh Chengamaraju.

**Formal analysis:** Muhammad Yousaf, Merlin Marry Thomas.

**Investigation:** Salah Almughalles.

**Methodology:** Muhammad Yousaf.

**Project administration:** Muhammad Yousaf, Merlin Marry Thomas.

**Resources:** Muhammad Yousaf, Merlin Marry Thomas.

**Supervision:** Muhammad Yousaf, Merlin Marry Thomas, Mohamad Yahya Khatib.

**Validation:** Salah Almughalles, Ahmad Alharafsheh, Mona Babikir, Dinesh Chengamaraju.

**Visualization:** Muhammad Yousaf, Merlin Marry Thomas, Mansoor Ali Hameed.

**Writing – original draft:** Muhammad Yousaf, Merlin Marry Thomas, Ahmad Alharafsheh, Irfan Varikkodan, Ali Waseem.

**Writing – review & editing:** Muhammad Yousaf, Merlin Marry Thomas, Mansoor Ali Hameed, Mohamad Yahya Khatib.
